# Effects of Inhibitors of the Activity of the Circulating Renin–Angiotensin System on the Growth and Proliferation of Endometrial Cancer Cells

**DOI:** 10.3390/ijms262210968

**Published:** 2025-11-12

**Authors:** Sarah J. Delforce, Riazuddin Mohammed, Tess L. Symington, Yu Wang, Nicole M. Verrills, Eugenie R. Lumbers, Kirsty G. Pringle

**Affiliations:** 1 School of Medicine and Public Health, College of Health, Medicine and Wellbeing, University of Newcastle, Newcastle, NSW 2308, Australia; sarah.delforce@newcastle.edu.au; 2 School of Biomedical Sciences and Pharmacy, College of Health, Medicine and Wellbeing, University of Newcastle, Newcastle, NSW 2308, Australiatess.symington@uon.edu.au (T.L.S.); nikki.verrills@newcastle.edu.au (N.M.V.); e.lumbers@newcastle.edu.au (E.R.L.); 3 Cancer Detection and Therapy Program, Hunter Medical Research Institute, New Lambton Heights, NSW 2305, Australia; 4 Women’s Health Research Program, Hunter Medical Research Institute, New Lambton Heights, NSW 2305, Australia; 5 R&D, Beroni Group Ltd., Sydney, NSW 2000, Australia

**Keywords:** renin–angiotensin system, endometrial cancer, renin inhibitors, ACE inhibitors, angiotensin receptor blockers

## Abstract

Endometrial cancers increase expression of the renin–angiotensin system (RAS). This study aimed to determine if inhibiting the RAS would reduce the viability and proliferation of endometrial cancer cells. The expression of RAS genes was measured in three endometrial epithelial adenocarcinoma cell lines (Ishikawa, HEC-1-A, AN3CA). Ishikawa cells had the highest expression of *REN*, *ACE*, and *AGTR1* mRNA. AGT mRNA and protein levels were most abundant in HEC-1-A cells. We then determined the effects of drugs that inhibit the action of renin (VTP-27999 and aliskiren) or angiotensin-converting enzyme (perindoprilat) or block the angiotensin II type 1 receptor (losartan and telmisartan). Overall, VTP-27999, aliskiren, perindoprilat, and losartan had minimal effects on cell viability in all three cell lines, and combinations of these drugs did not have any effect. Telmisartan (a dual angiotensin receptor blocker and PPAR-γ agonist) significantly reduced the viability of all three cell lines and reduced the proliferation of both Ishikawa and AN3CA cells. Telmisartan was more effective than troglitazone (PPAR-γ agonist) in Ishikawa and HEC-1-A cells. RAS inhibitors were most effective in Ishikawa cells, which had the highest levels of RAS expression. Therefore, levels of RAS expression in endometrial cancers might indicate the potential efficacy of RAS drugs.

## 1. Introduction

Endometrial cancer is the most common gynecological malignancy [1]. Risk factors for endometrial cancer include obesity, hypertension, and diabetes, all of which are associated with increased activity of the renin–angiotensin system (RAS) [2]. The circulating RAS consists of a cascade of enzymes that begins with renin acting on angiotensinogen (the sole source of angiotensin peptides) [3], to produce the biologically active peptides. Angiotensin II (Ang II) is the major end-product of the RAS, playing an important role in the regulation of blood pressure and water and electrolyte balance. Drugs that block the formation of Ang II or its interaction with the Ang II type 1 receptor (AT_1_R) are used widely in the treatment of hypertension, heart failure, and diabetic nephropathy [4,5,6].

**Figure 1:**
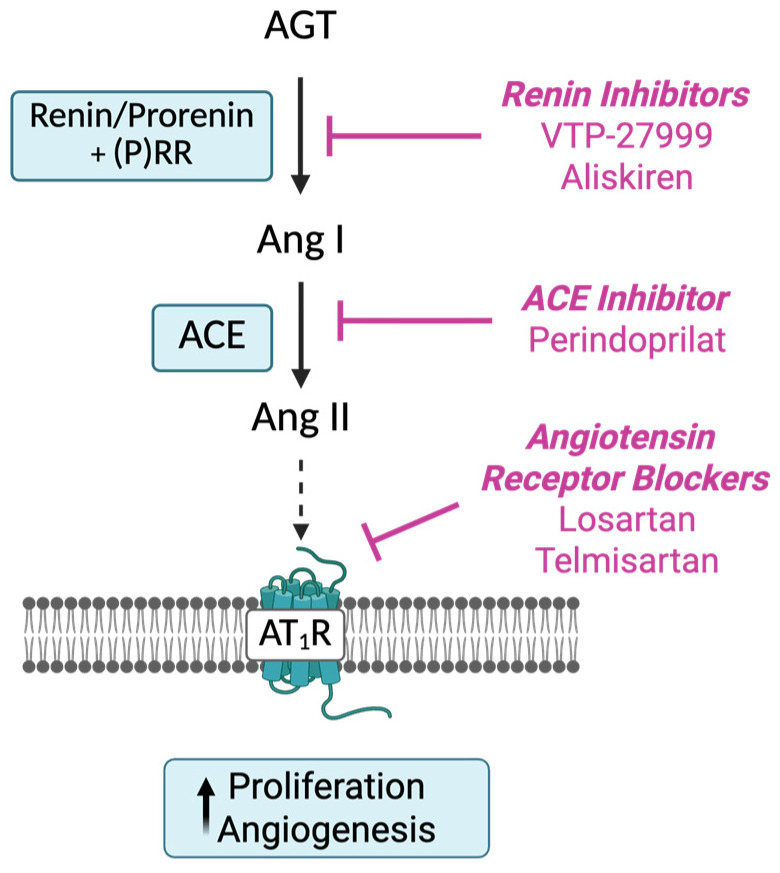
Sites of action of drugs acting on the RAS cascade. Renin inhibitors, including VTP-27999 and aliskiren, act directly on renin/prorenin to prevent the production of angiotensin (Ang) I from angiotensinogen (AGT). Angiotensin-converting enzyme inhibitors (ACEIs) act by inhibiting the conversion of Ang I to Ang II, resulting in the reduced production of Angiotensin (Ang) II, the main biologically active peptide in the cascade. Ang II receptor blockers (ARBs) act directly to inhibit activation of the Ang II type 1 receptor (AT_1_R). Created in BioRender. Pringle, K. (2025) https://BioRender.com/0xqzg94.

As well as the circulating RAS (Figure 1), there are, in tissues, ‘protease Ang II-producing systems’, which include prorenin (precursor of renin). Tissue RASs appear to play a role in cell growth, differentiation, and angiogenesis [7]. Since prorenin, the inactive precursor of renin, is the only form of renin produced in tissues, it has to be activated by removal of a 28-amino-acid pro-segment that covers its catalytic cleft by tissue proteases or by uncovering the catalytic cleft by binding to the (pro)renin receptor [8]. The decapeptide Ang I, produced by the action of renin, is cleaved by dipeptidase, an angiotensin-converting enzyme (ACE), to produce the biologically active octapeptide Ang II [9].

There is some evidence that long-term use of anti-hypertensives that reduce the activity of the RAS, such as ACE inhibitors (ACEIs) and angiotensin receptor blockers (ARBs), reduces the risk of some cancers, particularly female-specific cancers [10]. A more recent study has, however, shown that RAS inhibitors are associated with a decreased risk of cervical and ovarian cancer but an increased endometrial cancer risk [11]. Thus, the epidemiological associations are still under debate [12]. Previous laboratory studies showed that ARBs inhibited the growth of several cancers both in vivo and in vitro, including colon [13], gastric [14,15,16], breast [17,18], and endometrial cancers [19]. Watanabe et al. showed that endometrial cancer cells treated with the major effector peptide of the RAS, Ang II, had increased vascular endothelial growth factor (*VEGF*) expression and endothelial cell migration [20]. It should be noted that some ARBs, such as telmisartan, have additional actions including acting as a partial agonist of peroxisome proliferator-activated receptor gamma (PPAR-γ) [21].

Key genes controlling the synthesis and activity of RAS components like prorenin (*REN*), angiotensinogen (*AGT*), AT_1_R (*AGTR1*), and angiotensin converting enzyme (*ACE*), as well as Ang II, are present in the normal endometrium and show cyclical changes in levels of expression [22,23,24,25,26]. They are also abundantly expressed in endometrial cancer tissues and could play a role in cancer growth and angiogenesis [27,28,29,30,31]. Therefore, drugs that act on the RAS and that are used to treat hypertension could be repurposed and used in the control of growth and spread of endometrial cancer (Figure 1).

Since we had shown that the RAS is overexpressed in endometrial cancers [27], we measured expression of RAS genes (*REN*, *AGT*, *ACE*, and *AGTR1*) in three endometrial cancer cell lines and determined if cell viability and cell proliferation were inhibited by drugs that act to block the activity of the RAS at sites shown in Figure 1. These were the renin inhibitors (aliskiren and VTP-27999), a drug that inhibits ACE and blocks the formation of Ang II (perindoprilat), and drugs that block the interaction between Ang II and its AT_1_R (losartan and telmisartan).

## 2. Results

### 2.1. RAS mRNA and Protein Levels in Endometrial Cancer Cell Lines

**Figure 2:**
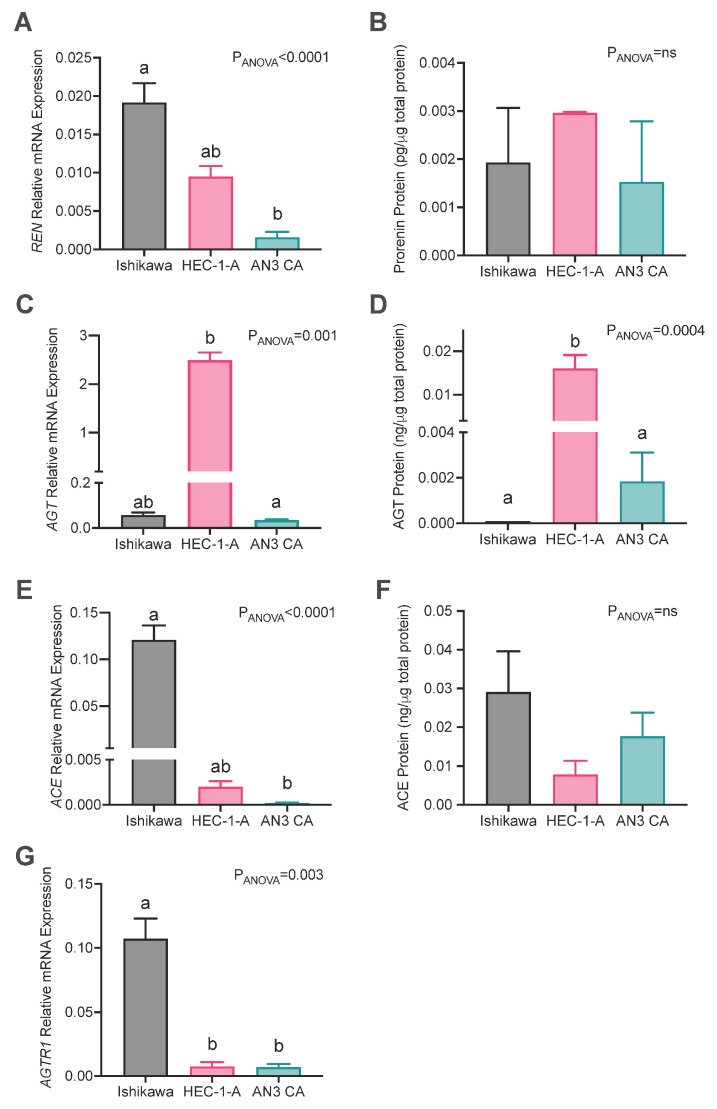
Expression of RAS genes in endometrial cancer cell lines. Abundances of (**A**,**B**) prorenin (*REN*), (**C**,**D**) angiotensinogen (*AGT*), (**E**,**F**) angiotensin-converting enzyme (*ACE*) mRNA and protein levels, and (**G**) angiotensin II type 1 receptor (*AGTR1*) mRNA abundances in Ishikawa, HEC-1-A, and AN3CA cells. Data are expressed as the mean ± SEM. Different superscript letters denote significant differences between cell lines. mRNA: N = 3 experiments performed independently with technical replicates. Protein abundance: N = 3–6 independent experiments.

Three endometrial cancer cell lines (Ishikawa, HEC-1-A, and AN3CA) were cultured, and RAS mRNA abundances and levels of secreted RAS proteins were measured. There were significant differences between these three cell lines in their expression of RAS genes and proteins (Figure 2).

The expression of *REN* mRNA was most abundant in Ishikawa cells, which had significantly higher levels of *REN* mRNA than AN3CA cells (*p* = 0.003) but not HEC-1A cells (Figure 2A). Conversely, prorenin protein levels in cell culture supernatants were similar in all three cell lines (Figure 2B). *AGT* mRNA and protein levels were abundant in HEC-1-A cells (Figure 2C,D) and were greater than those in AN3CA (mRNA: *p* = 0.004; protein: *p* = 0.038) and Ishikawa cells, although not significantly at the mRNA level for Ishikawa cells (mRNA: *p* = 0.124; protein: *p* = 0.003). *ACE* mRNA expression was highest in Ishikawa cells compared with HEC-1A and AN3CA, although the difference between Ishikawa and HEC-1A cells was not statistically significant (*p* = 0.17 and *p* = 0.002, respectively; Figure 2E). Conversely, there was no difference in ACE protein levels in cell culture supernatants from the three cell lines (Figure 2F). *AGTR1* expression was significantly higher in Ishikawa cells compared with both HEC-1-A and AN3CA cells (*p* = 0.025 and 0.038, respectively, Figure 2G). Thus, overall Ishikawa had the greatest levels of expression of *REN* and *ACE* and HEC-1-A had the highest expression of *AGT*. On the other hand, only in HEC-1-A cells were protein levels greater than that measured in other cell lines, specifically AGT.

### 2.2. Effect of Blockade of the RAS on Cell Viability and Proliferation Rates

#### 2.2.1. Effect of Renin Inhibitors VTP-27999 and Aliskiren

Treatment with VTP-27999 caused a minimal reduction in the cell viability of Ishikawa and HEC-1-A cells by an average of 7–13% at doses of 10 and 100 μM (Supplementary Figure S1). Treatment with aliskiren reduced cell viability in Ishikawa cells by an average of 20% (Supplementary Figure S1). Effects were only significant with high doses (100 μM). There were no effects on the viability of AN3CA cells (Supplementary Figure S1).

#### 2.2.2. Effects of Angiotensin Receptor Blockers (ARBs) Losartan and Telmisartan on Cell Viability and Cell Proliferation

**Figure 3:**
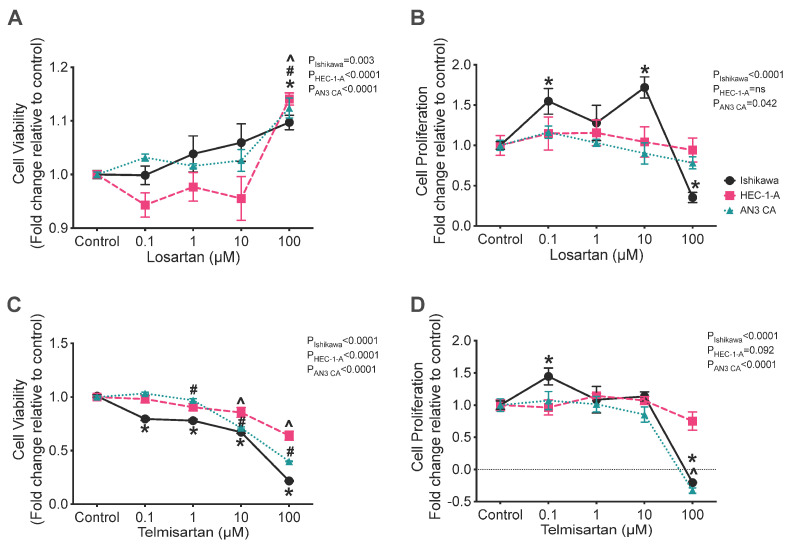
Effect of angiotensin receptor blockers (losartan and telmisartan) on the cell viability (**A**,**B**) and rate of cell proliferation (**C**,**D**) in Ishikawa (black), HEC-1-A (pink), and AN3CA (blue) cells. (**A**) Treatment with 100 μM of losartan significantly enhanced the viability of Ishikawa, HEC-1-A, and AN3CA compared with that of their respective vehicle controls (*, *p* = 0.003, #, *p* = 0.0009, and ^, *p* < 0.0001, respectively). (**B**) In Ishikawa cells, treatment with 0.1 or 10 μM of losartan significantly enhanced the proliferation of Ishikawa cells compared with that of vehicle control cells (*: *p* = 0.018 and 0.002, respectively). At 100 μM of losartan, the proliferation of Ishikawa cells was significantly reduced compared with that of vehicle control cells (*: *p* = 0.005). (**C**) Treatment with telmisartan significantly reduced cell viability in Ishikawa, HEC-1-A, and AN3CA cells at 10 and 100 μM (*, both *p* < 0.0001, #, *p* = 0.001 and <0.0001, and ^, both *p* < 0.0001, respectively). At 1 μM of telmisartan, the viability of Ishikawa and HEC-1-A cells was reduced compared with that of vehicle control cells (*, *p* < 0.0001 and #, *p* = 0.045, respectively). Ishikawa cell viability was also significantly reduced with treatment with 1 μM of telmisartan (*: *p* < 0.0001). (**D**) The cell proliferation of Ishikawa cells was enhanced by treatment with 1 μM of telmisartan compared with that of vehicle control cells (*: *p* = 0.042). Treatment with telmisartan at 100 μM significantly reduced the cell proliferation of Ishikawa and AN3CA cells (*, *p* < 0.0001 and ^, *p* < 0.0001, respectively). In HEC-1-A cells, proliferation was unaffected by treatment with telmisartan. Results are expressed as mean fold change relative to respective vehicle control cells ± SEM. N = 3 independent experiments in triplicate.

Treatment with losartan had a small stimulatory effect on the viability of Ishikawa, AN3CA, and HEC-1-A cells (Figure 3A) at a dose of 100 μM (*p* = 0.003, 0.0009, and <0.0001, respectively) and markedly stimulated the proliferation rate of Ishikawa cells with doses of 0.1 and 10 μM (*p* = 0.018 and 0.002, respectively). However, treatment with 100 μM of losartan was associated with a 65% reduction in Ishikawa cell proliferation (Figure 3B; *p* = 0.005).

Treatment with telmisartan caused a consistent reduction in the viability of all three cell lines (Figure 3C). Treatment with telmisartan at a concentration of 0.1, 1, 10, or 100 μM significantly reduced the viability of Ishikawa cells compared with the control (Figure 3C; all *p* = 0.0001) and treatment with 1, 10, or 100 μM of telmisartan also reduced the viability of HEC-1-A cells (Figure 3C; *p* = 0.045, 0.001, and 0.0001, respectively). In AN3CA cells, cell viability was also significantly reduced by treatment with 10 or 100 μM of telmisartan (Figure 3C; both *p* = 0.0001).

Additionally, treatment with telmisartan significantly altered the rates of cell proliferation of both Ishikawa and AN3CA cells, although it had no effect on the proliferation of HEC-1-A cells (Figure 3D). In Ishikawa cells, telmisartan had a biphasic effect on their rate of proliferation; 0.1 μM of telmisartan was associated with a 45% increase in the rate of cell proliferation (Figure 3D; *p* = 0.04), whereas 100 μM of telmisartan was associated with a 120% reduction in cell proliferation, which was indicative of cell death (Figure 3D; *p* = 0.0001). Similarly, 100 μM of telmisartan was associated with a significant reduction in cell proliferation of 130% in AN3CA cells (Figure 3D; *p* = 0.0001).

#### 2.2.3. Comparison of the Effects of Telmisartan with a PPAR-γ Agonist (Troglitazone) and the Efficacy of Combination Treatment with Telmisartan and Troglitazone on Cell Viability and Proliferation

Since telmisartan, which is both an ARB and a PPAR-γ agonist, was effective in inhibiting cell viability and proliferation of all three cell lines, we determined if its effects depended on the fact that it is a PPAR-γ agonist as well as an ARB. Therefore, the three cell lines were cultured with troglitazone or telmisartan alone and both in combination.

**Figure 4:**
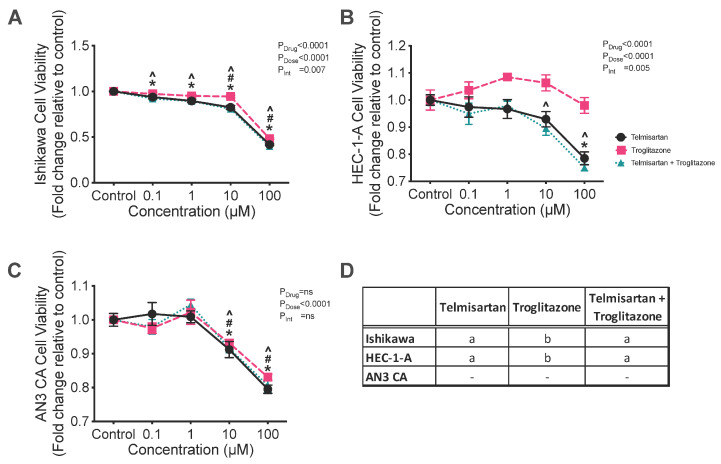
Effect of the combination of telmisartan, an ARB and partial PPAR-γ agonist, and troglitazone, a PPAR-γ agonist, on cell viability in (**A**) Ishikawa, (**B**) HEC-1-A, and (**C**) AN3CA cells. (**A**) Ishikawa cell viability was reduced by treatment with 0.1, 1, 10, or 100 μM of telmisartan alone and telmisartan + troglitazone (*, *p* = 0.0090, <0.0001, <0.0001, and <0.0001 and ^, *p* = 0.0006, <0.0001, <0.0001, and <0.0001, respectively). Troglitazone reduced the viability of Ishikawa cells at 10 and 100 μM (#: *p* = 0.022 and <0.0001, respectively). (**B**) Treatment with telmisartan significantly reduced the viability of HEC-1-A cells at 100 μM (*: *p* < 0.0001). Combined treatment with telmisartan + troglitazone reduced the viability of HEC-1-A cells at 10 and 100 μM (^: *p* = 0.035 and <0.0001). (**C**) Treatment with telmisartan alone, troglitazone alone, or telmisartan + troglitazone reduced the viability of AN3CA cells at 10 and 100 μM (*, *p* = 0.007 and <0.0001, #, *p* = 0.050 and <0.0001, and ^, *p* = 0.01 and <0.0001, respectively). (**D**) Different letters denote significant differences in the drug treatments within each cell line. Results are expressed as mean fold change relative to respective vehicle control cells ± SEM. N = 3 independent experiments in triplicate.

Troglitazone alone was the least effective treatment; both telmisartan alone and the combination of telmisartan + troglitazone were more effective at reducing the cell viability of Ishikawa cells compared with troglitazone alone (Figure 4D; *p* < 0.0001). The viability of Ishikawa cells was reduced by treatment with 0.1, 1, 10, or 100 μM of both telmisartan alone and telmisartan + troglitazone by an average of 10–60% (Figure 4D, all *p* < 0.01). Troglitazone alone reduced the cell viability of Ishikawa cells by an average of 50% at 10 and 100 μM (Figure 4A, *p* = 0.022 and <0.0001, respectively).

Treatment with telmisartan alone and telmisartan + troglitazone in combination were also more effective at reducing the viability of HEC-1-A cells compared with troglitazone alone (Figure 4D; both *p* < 0.0001). The combined treatment of telmisartan + troglitazone significantly reduced the viability of HEC-1-A cells at 100 μM by an average of 25% (Figure 4B; both *p* < 0.0001). Combined treatment with telmisartan + troglitazone also reduced the viability of HEC-1-A cells at 10 μM by an average of 10% (*p* = 0.035, Figure 4B).

In AN3CA cells, treatment with telmisartan alone, troglitazone alone, or telmisartan + troglitazone reduced the viability of AN3CA cells at 10 and 100 μM by approximately 10 and 20%, respectively (Figure 4C; all *p* < 0.05). There were no significant differences between these three treatments (Figure 4D).

#### 2.2.4. Combined Effect of Aliskiren and an ACEI (Perindoprilat) or an ARB (Losartan) on Cell Viability

Since the effects of the inhibitors of the circulating RAS were limited in their capacity to reduce cell viability or inhibit cell proliferation, it was decided to see if the combination of a renin inhibitor with either an ACEI or with an ARB was more effective.

The combination treatment of aliskiren + perindoprilat was significantly more effective at reducing cell viability compared with either aliskiren or perindoprilat alone in HEC-1-A cells (Supplementary Figure S2), neither of which had any effect on cell viability. The combined treatment of aliskiren + perindoprilat significantly reduced the cell viability of HEC-1-A cells at 10 and 100 μM by approximately 10–15% (Supplementary Figure S2; *p* = 0.0004 and <0.0001).

Overall, treatment of AN3CA cells with inhibitors either alone or in combination had no inhibitory effect on the viability of AN3CA cells and had minimal enhancing effects (<5% change from control, Supplementary Figure S2).

The combination of aliskiren and losartan had no synergistic effect on the viability of Ishikawa, HEC-1-A, or AN3CA cells (Supplementary Figure S3). However, in Ishikawa cells, the combination of aliskiren and losartan mitigated the effect of aliskiren alone (Supplementary Figure S3).

## 3. Discussion

We have shown that genes controlling the expression of key components of the RAS cascade were expressed in the Ishikawa, HEC-1-A, and AN3CA endometrial cancer cell lines. Overall, Ishikawa cells appeared to express *REN*, *ACE*, and *AGTR1* mRNAs in the greatest abundance. Therefore, it was not surprising that any effects of RAS-blocking drugs on cell viability and proliferation were most evident in the Ishikawa cells. Overall, the AN3CA cell line was most resistant to any inhibitory effects of the drugs studied on cell viability and/or proliferation.

Low levels of mRNA expression of *REN* in all three cell lines (Figure 2, as determined by a qPCR cycle threshold > 32), as well as low levels of soluble prorenin, may indicate that *REN* was not expressed in amounts sufficient to activate the RAS. This could account for the low efficacy of the two renin inhibitors studied, i.e., aliskiren and VTP-27999 (Supplementary Figure S1). We have previously shown that endometrial adenocarcinomas express prorenin mRNA and protein abundantly [27], so these low levels of *REN* mRNA were unexpected. We have also shown that there is a wide range of *REN* expression in both the cancerous endometrium and adjacent normal endometrium [27]. Therefore, while the drugs are ineffective in inhibiting cancer growth in established cancer cell lines, they may block the increased activity of the RAS in the pre-cancerous stages of endometrial dysfunction associated with endometrial hyperplasia and estrogen stimulation [32]. This may also indicate that *REN* mRNA is primarily produced in the stroma of the endometrium, rather than the glandular epithelium. Thus, these drugs should be assessed in culture models containing both stromal and glandular cells.

Interestingly, the expression of RAS components tends to correlate negatively with the histological grade the cell line represents. That is, Ishikawa cells, which expressed the highest level of most RAS components, are histologically representative of grade 1 tumours [33], whereas the AN3CA cell line, which expressed the lowest levels of RAS components, is histologically representative of grade 3 tumours (metastatic undifferentiated) [34]. This indicates that the RAS may be inactivated as cancer cells differentiate. This was also shown by Piastowska-Ciesielska et al. who found that the expression of *AGTR1* mRNA and protein levels was highest in grade 1 tumours compared with grade 3 tumours [28]. Thus, it is possible that at higher grades, the RAS is no longer regulating endometrial cancer cell growth and RAS-inhibiting drugs are likely to be ineffective. It is also possible that RAS inhibitors may be effective in the prodromal phase of disease, such as during endometrial hyperplasia, prior to the development of endometrial cancer.

The three cell lines used in the study have also been reported to have different levels of estrogen receptors [35] and differing responses to estrogen [36]. Ishikawa cells have high expression of estrogen receptor (ER)-α and ER-β [35] and are responsive to estrogen treatment [36], while the expression of these receptors is low in HEC-1-A and AN3CA cells [35]. Since many components of the RAS are stimulated by estrogens, it is perhaps not surprising that expression of RAS is highest in Ishikawa cells, which have high levels of the estrogen receptors [35] and are estrogen-responsive [36]. Given the different expression of RAS components and estrogen receptors in the cell lines, as well as the interactions between estrogen and the RAS, future studies should assess the impact of estrogens on RAS expression in these cells and the effects of the RAS antagonists in the presence of estrogens.

It is well established that estrogens can cause endometrial hyperplasia and pre-cancerous changes in the endometrium. This is particularly noted in patients taking tamoxifen (a selective estrogen receptor modulator) as an adjunct therapy for breast cancer. Patients undergoing tamoxifen treatment have a significantly increased risk of developing endometrial hyperplasia and a 1.5–6.9-fold increased risk of developing endometrial cancer [37]. Currently, no studies have looked at the expression of the endometrial RAS in women taking tamoxifen for the treatment of breast cancer. Future studies should therefore be undertaken to examine the endometrial RAS in these patients, as RAS inhibitors may be effective at reducing endometrial hyperplasia and the progression to endometrial cancer.

In most instances, the reduction in cell viability and/or proliferation caused by the RAS antagonists was minimal and significant reductions in cell viability and cell growth only occurred when high concentrations of drugs were used. Specifically, treatment with high doses of a renin inhibitor, VTP-27999 or aliskiren, was minimally effective at inhibiting cell viability and proliferation in Ishikawa and HEC-1-A cells and did not affect AN3CA cells. In addition, perindoprilat, an ACE inhibitor, did not have any effect on the viability of any of the three endometrial cancer lines. To the best of our knowledge, this is the first study to examine the effect of renin inhibitors (aliskiren and VTP-27999) and ACE inhibitors (perindopril) in endometrial cancer cells. Surprisingly, at high concentrations, losartan significantly increased the viability of all three cancer cell lines. This finding is similar to that reported by Koyama et al., who also showed that losartan increased the viability of endometrial cancer cells [19]. Contrastingly, the rate of proliferation of Ishikawa cells was significantly reduced when treated with 100 μM losartan. Since aliskiren, VTP-27999, perindopril, and losartan only reduced cell viability and proliferation to a limited extent (~10–15%) and high doses were required to have any effect, only telmisartan would likely be of clinical value as an adjunct treatment for endometrial cancer. These conclusions are supported by the fact that the combination of drug treatments used in this study had no additive effect.

Overall, telmisartan, the combined ARB and PPAR-γ agonist [21], appeared to be the most effective inhibitor of cell viability in all three cell lines (Figure 3 and Figure 4). Telmisartan’s most significant effects were on Ishikawa cells, which expressed *REN*, *ACE*, and *AGTR1* to a greater extent than the other cell lines. These three genes are the key regulators of Ang II production and signalling. It should be noted that in a previous study, we found an association between the prevalence of endometrial cancer and single nucleotide polymorphisms (SNPs) associated with increased expression of AT_1_R (rs5186) and ACE (rs4291 and rs4292) [38]. We also showed that the mRNA expression of *AGTR1* and *ACE* were significantly higher in endometrial cancer tissue, further indicating the potential for increased Ang II/AT_1_R signalling in endometrial cancer [27]. The efficacy of telmisartan in reducing the viability and proliferative capacity of endometrial cancer cell lines is consistent with results obtained by Koyama et al. [19]. The effects of telmisartan, in part, must be a result of AT_1_R inhibition as troglitazone, a sole PPAR-γ, alone was not as effective at reducing cell viability compared with telmisartan. These results are similar to those observed in human colon cancer cells [13].

Angiogenesis is essential for tumour growth and spread. Activation of AT_1_R by Ang II stimulates the secretion of VEGF from endometrial cancer cell lines, including HEC-1-A [20]. VEGF is a powerful angiogenic factor, which promotes tumour angiogenesis [39]. Recently, it has been shown that aerobic exercise induced the ACE/Ang II/AT_1_R/VEGF axis in skeletal muscle and increased the capillary-to-fibre ratio by inhibiting miRNAs miR-27a and b. It was further shown that losartan partially reversed these changes through effects on miR-27a and b [40]. Such an angiogenic pathway in tumorigenesis has not been demonstrated, yet the hypoxia induced by the metabolic demand of rapidly dividing pre-cancerous lesions could induce a similar response to that seen in exercising skeletal muscle. This merits its investigation in endometrial hyperplasia and endometrial cancer.

In conclusion, some of the drugs that limit the formation and actions of Ang II via the AT_1_R modestly reduce the viability and proliferation of endometrial cancer cells. This study did not examine the effects of these drugs on angiogenesis nor on cell migration, two other properties of cancer cells that promote malignancy. Also, some of the effects we observed could be off-target actions of the drugs. However, their increased efficacy in the cell line that grew most vigorously and had the highest level of expression of the renin–Ang II/AT_1_R pathway (Ishikawa) suggests that their effects on cell viability and proliferation were at least partially related to their inhibitory effects on the RAS in this tumour cell line.

## 4. Materials and Methods

### 4.1. Cell Culture

Three endometrial epithelial adenocarcinoma cell lines were used for this study; these were Ishikawa (confirmed by genotyping), AN3CA, and HEC-1-A cells (American Type Culture Collection, Manassas, VA, USA). Ishikawa and AN3CA cells were cultured in minimum essential medium (MEM; Sigma Aldrich, St. Louis, MO, USA) supplemented with 5% heat-inactivated fetal bovine serum (HI-FBS; Bovogen Biologicals, Keilor East, VIC, Australia) and 1% antibiotic–antimycotic (Thermo Fisher Scientific, Waltham, MA, USA). HEC-1-A cells were cultured in McCoy’s 5A medium (Sigma Aldrich) supplemented with 10% HI-FBS (Bovogen Biologicals), 1% antibiotic–antimycotic, and 2 mM L-glutamine (Thermo Fisher Scientific). For gene expression studies, cells were seeded at a density of 1.5 × 10^5^ in 6-well plates in 2 mL of incubation medium and allowed to settle overnight. After 24 h, the incubation medium was changed, and cells were cultured for 48 h. Cells were then harvested and snap frozen in liquid nitrogen and stored at −80 °C for subsequent mRNA analyses.

### 4.2. Semi-Quantitative Real-Time Reverse Transcriptase Polymerase Chain Reaction (qPCR)

Total RNA was isolated using the RNeasy mini kit according to the manufacturer’s instructions (Qiagen, Hilden, Germany). RNA samples were DNase-treated (Qiagen). RNA quantity was assessed using the Nanodrop spectrophotometer and RNA quality was determined by agarose gel electrophoresis. RNA was then reverse-transcribed using a Superscript III RT kit with random hexamers (Invitrogen, Waltham, MA, USA).

qPCR was performed with a 7500 Real Time PCR System (Applied Biosystems, Waltham, MA, USA) using SYBR Green for detection. Each reaction contained 5 μL of SYBR Green PCR master mix (Applied Biosystems), primers [27], cDNA reversed-transcribed from 10 ng of total RNA, and water to 10 μL. Genes examined were *REN*, *ATP6AP2 AGT*, *ACE*, and *AGTR1*. The geometric average of the three housekeepers was measured. These housekeepers were β-actin (*ACTB*; [27]), 18s ribosomal RNA (*RNA18S1*; *Fwd*: *GTAACCCGTTGAACCCCATT*; *Rev*: *CCATCCAATCGGTAGTAGCG*), and tyrosine 3-monooxygenase/tryptophan 5-monooxygenase activation protein zeta (*YWHAZ*; *Fwd*: *CCTGCATGAAGTCTGTAACTGAG*; *Rev*: *GACCTACGGGCTCCTACAACA*), which were stably expressed between cell types. Messenger RNA abundance was calculated as described previously, using the 2^−ΔΔCT^ method and expressed relative to the mean of *ACTB*, *RNA18S1*, and *YWHAZ* and a calibrator sample (a term placental sample collected upon elective cesarean section) [19].

### 4.3. ELISAs

Total protein was isolated from cell culture media by acetone precipitation for both prorenin and AGT ELISA, as previously described [41]. ACE protein from cell culture media was run undiluted. Acetone precipitation was completed by combining the medium supernatant with three times the volume of chilled acetone (−20 °C, UNIVAR, Downers Grove, IL, USA). Samples were then incubated at −20 °C for 4 h before being centrifuged at 14,000× *g* for 10 min at 4 °C. The precipitated proteins were re-solubilised in RIPA buffer and quantified by BCA assay (Thermo Fisher Scientific).

The levels of prorenin, AGT, and ACE were measured by commercially available ELISA kits (Prorenin ELISA, Abcam, Cambridge, UK; AGT and ACE Duoset ELISAs, R&D Systems, Minneapolis, MN, USA) according to the manufacturer’s instructions. As all samples were assayed on one plate, there was no inter-assay variability. The intra-assay variability was 8.4% for prorenin, 4.3% for AGT, and 4.2% for ACE.

### 4.4. Assessment of Cell Viability

Effects of RAS inhibitors on cell viability was assessed using a Resazurin Assay. Cells were seeded in 100 μL of respective medium in 96-well plates and allowed to settle overnight. The cells were seeded at densities determined by individual cell growth rates. The cell densities for Ishikawa, HEC-1-A, and AN3CA were 1.5 × 10^2^ cells/well, 5 × 10^3^ cells/well, and 2.5 × 10^2^ cells/well, respectively. After 24 h the culture medium was replaced with medium containing a 0.1 μM, 1 μM, 10 μM, or 100 μM concentration of one of the following drugs:

(1) A renin inhibitor (either aliskiren or VTP-27999; Medchem Express);
(2) An ACE inhibitor (perindoprilat; Sigma Aldrich);
(3) An angiotensin receptor blocking drug (either losartan or telmisartan; Sigma Aldrich);
(4) A PPAR-γ agonist (troglitazone; Sigma Aldrich).

Or the medium contained a combination of the following (at the doses above):

(1) Aliskiren and perindoprilat;
(2) Aliskiren and losartan;
(3) Telmisartan and troglitazone.

Drug concentrations of 0.1–100 μM were chosen based on previously published studies investigating the effects of these drugs on cell proliferation in other cancer cell types [42,43,44,45,46,47].

At 43 h of culture, 5 h before the endpoint, 20 μL of Resazurin Reagent (BioVision, Milpitas, CA, USA, 0.15 mg/mL) was added to each well and the plates were returned to the incubator. At 48 h, fluorescence was measured at 590 nM emission using the FLUOStar OPTIMA (BMG Labtech, Offenburg, Germany) after 570 nM excitation. Data are expressed as the fold change (in relative fluorescence units (RFU)) relative to the average of the vehicle control for each experiment for each cell line.

### 4.5. Measurement of Cell Proliferation

The effects of RAS inhibitors on cell proliferation were measured using an xCELLigence RTCA DP instrument (ACEA Biosciences, Hangzhou, China). Sixteen-well E-plates (ACEA Biosciences) were used to study the rate of cell proliferation. Briefly, prior to beginning the experiment, 100 μL of medium was added to each well to allow the E-plate to achieve equilibrium. After 30 min, a background impedance reading was recorded. One hundred microliters of medium containing the densities of each cell type (described above) was added and the E-plates were inserted into the xCELLigence machine and left for 30 min to allow the cells to attach to the plate, after which readings commenced. After 24 h, the culture medium was replaced with fresh medium containing the drugs listed above. Cell proliferation was monitored by measuring the cell index (change in electrical impedance) every 15 min for 48 h. Data are expressed as the fold change in slope of the impedance trace over 48 h, relative to the average of the vehicle control for each experiment for each cell line assessed.

### 4.6. Statistics

All experiments were performed three times, with each dose being tested at least in duplicate. A Kruskal–Wallis test with Dunn’s multiple-comparisons test was used to determine differences in mRNA expression between cell lines and the effects of the drug treatments within each cell line. GraphPad Prism (Version 10.0) was used for all graphs and statistical analyses. Statistical significance was set at *p* < 0.05. If a sample had undetectable levels of mRNA expression as measured by qPCR, a value of 1 × 10^−5^, which was 100 times lower than the lowest value, was allocated so that it could be included in the analyses.

## 5. Conclusions

Overall, this is the first study to examine the effect of renin inhibitors on endometrial cancer cell lines. Levels of expression of the RAS genes may therefore be biomarkers for drug efficacy. Telmisartan consistently reduced cell proliferation and cell viability in all three endometrial cancer cell lines and was more effective than either a renin inhibitor, a sole ARB (losartan), or a sole PPAR-γ agonist. Future studies examining the efficacy of these RAS blockers in a mouse model of endometrial cancer will shed more light on the potential of RAS blockers to inhibit endometrial cancer growth and vasculogenesis.

## Data Availability

The original contributions presented in this study are included in the article/Supplementary Material. Further inquiries can be directed to the corresponding author.

## Supplementary Materials

The following supporting information can be downloaded at https://www.mdpi.com/article/10.3390/ijms262210968/s1.
