# Supplementary material for: Effects of Inhibitors of the Activity of the Circulating Renin–Angiotensin System on the Growth and Proliferation of Endometrial Cancer Cells
Source: Int J Mol Sci. 2025 Nov 12;26(22):10968. doi: 10.3390/ijms262210968 (PMC12652496; doi:10.3390/ijms262210968)
Supplement: Supplementary file 1 [file ijms-26-10968-s001.zip › ijms-3956333-supplementary.pdf]

SUPPLEMENTARY DATA

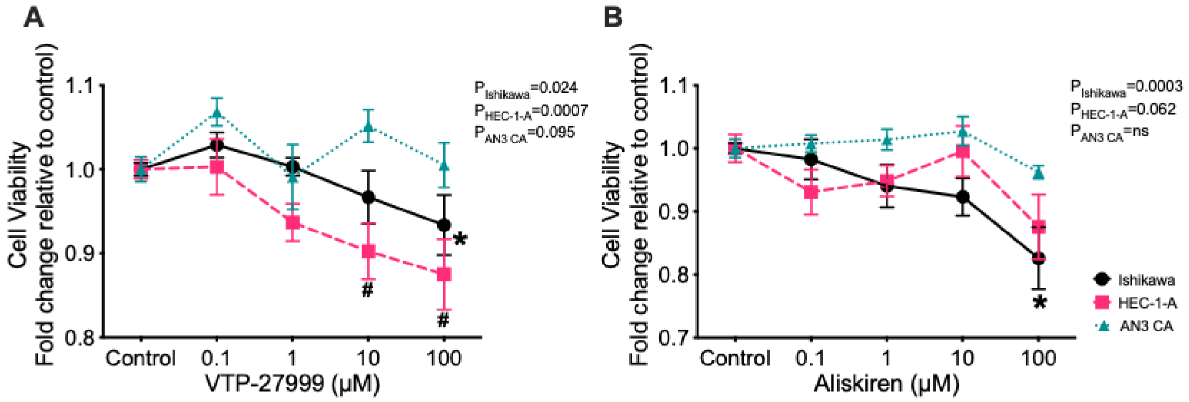

Supplementary Figure S1. *Effect of renin inhibitors, (A) VTP-27999, and (B) aliskiren, on cell viability in Ishikawa (black), HEC-1-A (pink) and AN3CA (blue) cells. Ishikawa cell viability was significantly inhibited by 100 µM of VTP-27999 and aliskiren compared with the vehicle control (*; P=0.04 and 0.0001, respectively). HEC-1-A cell viability was significantly inhibited by both 10 and 100 µM of VTP-27999 compared with vehicle control (#; P=0.02 and 0.001, respectively) and was unaffected by treatment with aliskiren. AN3CA cell viability was not affected by treatment with either VTP-27999 or aliskiren. Results are expressed as mean fold change relative to respective vehicle control ± SEM. N=3 independent experiments in triplicate.*

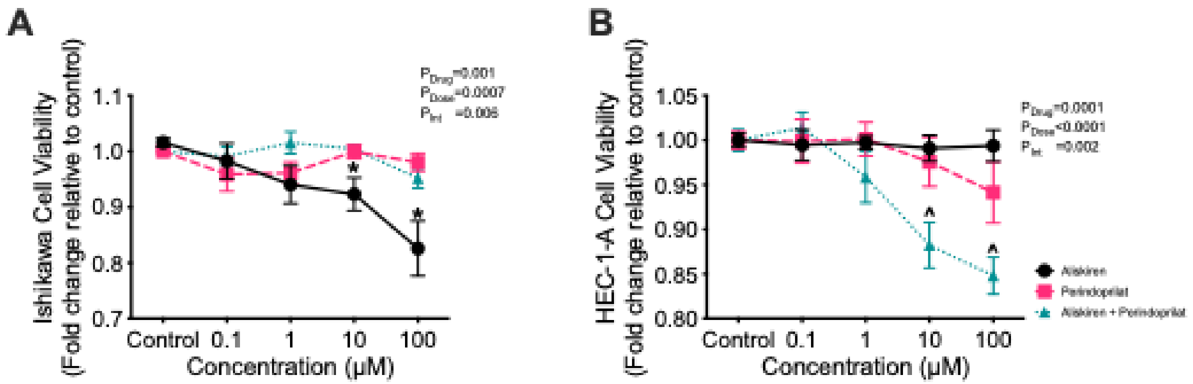

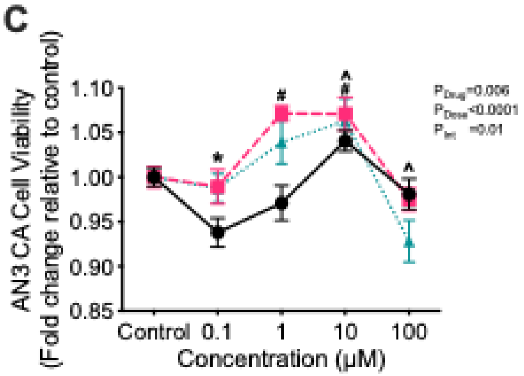

D

	Aliskiren	Perindoprilat	Aliskiren + Perindoprilat
Ishikawa	a	b	b
HEC-1-A	a	a	b
AN3 CA	a	b	ab

Supplementary Figure S2. *Effect of the combination of aliskiren, a renin inhibitor, and perindoprilat, an ACEI, on cell viability in (A) Ishikawa, (B) HEC-1-A, and (C) AN3CA cells. (A) Ishikawa cell viability was reduced by treatment with 10 and 100 µM of aliskiren alone (*; P=0.03 and <0.0001, respectively). (B) Combined treatment with aliskiren + perindoprilat significantly reduced the cell viability of HEC-1-A cells at 10 and 100 µM (^; P=0.0004 and <0.0001). (C) Treatment with aliskiren alone inhibited the cell viability of AN3CA cells at 0.1 µM (*; P=0.044). Treatment with perindoprilat alone enhanced cell viability of AN3CA cells at 1 and 10 µM (#; P=0.014 and 0.016, respectively). Combined treatment with aliskiren + perindoprilat significantly enhanced and inhibited cell viability of AN3CA cells at 10 and 100 µM, respectively (^; P= 0.040 and 0.014, respectively). (D) Different letters denote significant differences in the drug treatments within each cell line. Results are expressed as mean fold change relative to respective vehicle control ± SEM. N=3 independent experiments in triplicate.*

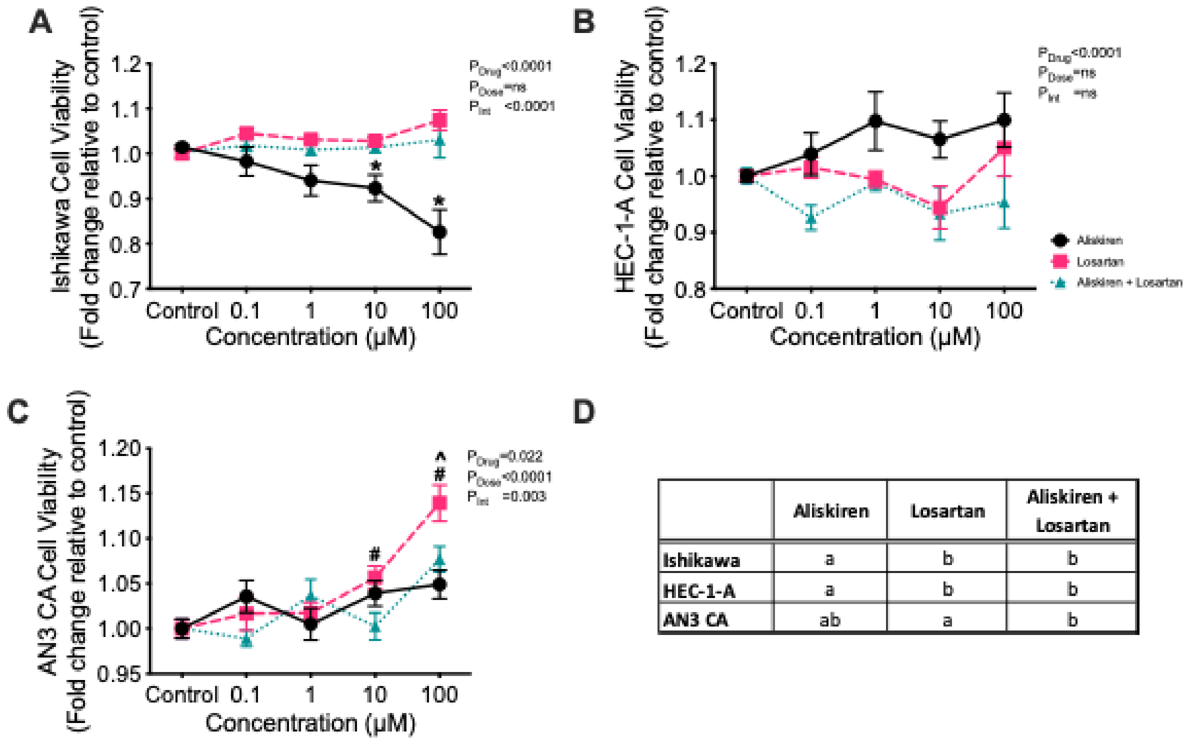

	Aliskiren	Losartan	Aliskiren + Losartan
Ishikawa	a	b	b
HEC-1-A	a	b	b
AN3 CA	ab	a	b

Supplementary Figure S3. *Effect of combination of aliskiren, a renin inhibitor, and losartan, an ARB, on cell viability in (A) Ishikawa, (B) HEC-1-A, and (C) AN3CA cells. Ishikawa cell viability was reduced by treatment with 10 and 100 µM of aliskiren alone (*; P=0.01 and <0.0001, respectively). HEC-1-A cell viability was unaffected by all treatments. Treatment with losartan alone, enhanced cell viability of AN3CA cells at 10 and 100 µM (#; P=0.03 and <0.0001, respectively). Combined treatment with aliskiren + losartan significantly enhanced cell viability at 100 µM (^; P=0.001). (D) Different letters denote significant differences in the drug treatments within each cell line. Results are expressed as mean fold change relative to respective vehicle control ± SEM. Three experiments were performed independently in triplicate.*
